# Characteristics of indeterminate QuantiFERON-TB Gold PLUS results in pediatric lupus nephritis at Beijing Children’s Hospital, 2023–2024

**DOI:** 10.1186/s12882-025-04652-9

**Published:** 2025-11-25

**Authors:** Yue Xi, Huiwen Zheng, Yonghong Wang, Yajie Guo, Jing Xiao, Feina Li, Hui Qi, Weiwei Jiao, Nan Zhou, Zhi Chen, Lin Sun

**Affiliations:** 1 Department of Nephrology, Beijing Children’s Hospital, Capital Medical University, National Center for Children’s Health, Beijing, China; 2 Laboratory of Respiratory Diseases, Beijing Pediatric Research Institute, Beijing Children’s Hospital, Capital Medical University, Beijing Key Laboratory of Core Technologies for the Prevention and Treatment of Emerging Infectious Diseases in Children, Key Laboratory of Major Diseases in Children, Ministry of Education, National Clinical Research Center for Respiratory Diseases, National Center for Children’s Health, Beijing, China

**Keywords:** Lupus nephritis, Interferon gamma release tests, Pediatrics, Latent tuberculosis

## Abstract

**Objectives:**

Tuberculosis screening in pediatric lupus nephritis (LN) patients presents unique diagnostic challenges. This study aimed to analyze the frequency of indeterminate QuantiFERON-TB Gold Plus (QFT-Plus) results among children with LN and the potential influence factors.

**Methods:**

A retrospective cohort study was conducted among patients under 18 years old with a confirmed diagnosis of LN screened for tuberculosis infection from January 2023 and August 2024. Demographic and clinical data were extracted from their electronic medical record, with categorical variables presented as frequencies and continuous variables as medians. Using SPSS 18.0 (Chicago, IL), we first performed univariate logistic regression to identify factors associated with indeterminate IFN-γ results (*P* < 0.05), then entered significant variables into multivariate models to determine independent predictors, reporting results as ORs, with statistical significance set at *P* < 0.05.

**Results:**

Of 111 patients with LN, 49 (44.14%) had indeterminate QFT-Plus results, with 43 (87.76%) due to positive control failure. In the 30-day period prior to the QFT-Plus test, 46 (41.44%) patients were treated with immunotherapy. Univariable logistic regression analysis indicated that the indeterminate group had significantly lower levels of hemoglobin, albumin, and alkaline phosphatase, fewer hospitalizations, and more frequent use of immunotherapy, and higher triglyceride and D-dimers levels. Multivariable logistic regression analysis revealed that triglycerides level (OR 1.847; 95% CI 1.195–2.856; *P* = 0.006) and immunotherapy use (OR 12.306; 95% CI 3.937–38.463; *P*<0.0001) were independently associated with increased risk of indeterminate QFT-Plus results. The area under the ROC curve for a triglycerides level of 2.155 mmol/L, and it was 0.751 for discriminating between indeterminate and determinate QFT-Plus results.

**Conclusions:**

Hospitalized patients with LN had a high rate of an indeterminate QFT-Plus result, predominantly in positive control failure. Besides, our data showed that the use of immunotherapy agents and high triglycerides level represent factors associated with indeterminate QFT-Plus results in our cohort of pediatric lupus nephritis patients.

**Supplementary Information:**

The online version contains supplementary material available at 10.1186/s12882-025-04652-9.

## Introduction

Tuberculosis (TB), a leading infectious cause of death globally, latently infects approximately one-quarter of the world’s population, of whom 10% will progress to active TB disease during their lifetime [1, 2]. The risk of reactivation from latent TB infection (LTBI), defined as *Mycobacterium tuberculosis* (MTB) infection without active symptoms, is substantially higher in infants (30%-40%) and children (10%-20%) than in adults [3]. Thus, early detection and initiation preventive treatment of LTBI are critical priorities for pediatric TB control.

Given the elevated risk of reactivation, comprehensive LTBI screening is widely recommended and represents a key standard of care for patients prior to initiating treatment with corticosteroids, immunosuppressants, or biologic agents [4–8]. This is particularly critical for patients with systemic lupus erythematosus (SLE), a multisystem autoimmune disorder characterized by immune dysregulation and pathogenic autoantibody production [9, 10]. Among the most severe clinical manifestations of SLE, lupus nephritis (LN) exhibits both higher prevalence and greater severity in pediatric populations compared to adults [11, 12]. Moreover, the aggressive immunosuppressive therapies in this population highlight the importance of implementing comprehensive screening for LTBI before pre-treatment, which can substantially reduce active TB incidence.

Current diagnostic approaches for pediatric LTBI screening rely on the tuberculin skin test (TST) and interferon-gamma release assays (IGRAs) [13–15]. The IGRA assay is an in vitro blood test that measures IFN-γ production by T cells following stimulation with MTB-specific antigens, which are unaffected by BCG vaccination status or most nontuberculous mycobacterial exposures [16]. The fourth-generation QuantiFERON-TB Gold Plus (QFT-Plus) IGRA assay utilizes an enzyme-linked immunosorbent assay (ELISA) platform featuring four distinct tubes: a negative-control (nil) tube for background IFN-γ quantification, a positive-control (mitogen) tube containing nonspecific T-cell stimulants to assess immune competence, and two antigen tubes (TB1 and TB2) incorporating novel CD8 + T-cell stimulating peptides to enhance detection sensitivity for MTB infection [17, 18]. Despite these technological advances, a significant limitation of IGRA persists in the form of indeterminate results, which occur more frequently in pediatric populations than adults, comprising accurate diagnosis of MTB infection [19]. While existing evidence indicates QFT-Plus generates fewer indeterminate results compared to its predecessor, QuantiFERON-TB Gold In-Tube (QFT-GIT), in pediatric populations [20, 21], the frequency of indeterminate results among children with LN and the potential influence of associated clinical and laboratory parameters remain poorly characterized. To address this knowledge gap, this study aims to analyze factors associated with indeterminate QFT-Plus results in pediatric LN patients, which may help optimize the timing of TB screening in patients requiring immunosuppressive therapy.

## Methods

### Participants

This retrospective study was performed at the Nephrology Department, Beijing Children’s Hospital, between January 2023 and August 2024. Eligible participants were under 18 years of age, with a confirmed diagnosis of LN based on clinical, endoscopic, radiographic, or histopathological criteria, and who had complete QFT-Plus test results and clinical data available. Patients with non-lupus nephritis-related kidney disorders (e.g., primary nephrotic syndrome, diabetic nephropathy) were excluded. Patients with incomplete clinical data, improper sample handling (e.g., delayed testing > 16 h), hemolysis or coagulation abnormalities affecting QFT-Plus results were also excluded.

### Analysis of clinical and laboratory characteristics

Clinical data included age, sex, disease duration, comorbid conditions, hospitalization frequency, medications and complications. Laboratory data included complete blood count: white blood cells (WBC), red blood cells (RBC), hemoglobin (Hb), platelets (PLT), neutrophils, lymphocytes, monocytes; blood biochemistry: albumin, C-reactive protein (CRP), albumin/globulin ratio (A/G ratio), urea, creatinine, total cholesterol, uric acid, alkaline phosphatase (ALP), aspartate aminotransferase (AST)/alanine aminotransferase (ALT), total bilirubin, cholinesterase, lactate dehydrogenase (LDH), triglycerides; erythrocyte sedimentation rate (ESR); coagulation: prothrombin time (PT), fibrinogen, thrombin time (TT), activated partial thromboplastin time (APTT), D-dimer, antithrombin III activity (AT-III); complement system: complement 3 (C3), complement 4 (C4); early kidney injury biomarkers: urinary IgG, urinary microalbumin, urinary transferrin, urinary α1-microglobulin, urinary β2-microglobulin, N-acetyl-β-D-glucosaminidase (NAG).Data were categorized by QFT-Plus results, with subsequent comparison of clinical and laboratory findings between determinate and indeterminate test result groups. For CRP, values below 10 mg/L are considered normal, while values equal to or above 10 mg/L are considered abnormal. The immunotherapy consisted of immunoglobulin (IVIg) or/and immunosuppressor or/and corticosteroids. Disease severity was assessed using the Systemic Lupus Erythematosus Disease Activity Index – Responder (SLEDAI-R) [22].

### QFT-plus assay

Following the manufacturer’s protocol, venous blood (3 mL) was collected into lithium heparin tubes and aliquoted into four separate QFT-Plus tubes. The tubes were incubated at 37 °C for 16–24 h, followed by centrifugation at 2000 × g for 15 min to harvest supernatants for IFN-γ quantification (IU/mL) via ELISA. IFN-γ levels in the antigen tubes that >10 IU/ml were classified as 10 IU/mL. Positive results were defined as either IFN-γ values of TB1-nil or TB2-nil ≥ 0.35 IU/mL, and ≥ 25% of the nil value; and negative when they were < 0.35 IU/mL. Nil value of >8.0 IU/mL and /or a mitogen minus nil tube value of <0.5 IU/mL were considered indeterminate. Positive and negative results were categorized as “determinate”.

### Statistical analysis

Categorical variables were expressed as frequencies and percentages, while continuous variables were presented as median and interquartile range (IQR). Univariate logistic regression analysis for all candidate variables were performed to identify factors associated with indeterminate IFN-γ results. Least Absolute Shrinkage and Selection Operator (LASSO) regression was employed for variable selection to effectively mitigate the risk of model overfitting. Additionally, non-collinear variables showing a significant association (*P* < 0.1), as well as clinically relevant predictors, were incorporated into the final multivariate logistic regression analysis. Results were reported as odds ratios (ORs) with corresponding 95% confidence intervals (CIs). All statistical analyses were performed using SPSS version 18.0 software (SPSS, Chicago, IL, USA).

## Results

### Patient characteristics

A total of 111 patients with LN underwent QFT-Plus testing were enrolled from 2023 to 2024, consisting of 2 (1.80%) positive, 60 (54.04%) negative and 49 (44.14%) indeterminate results. The median age of the study participants was 13 years and the majority were female (81.08%). The median frequency of hospitalization was 10 times. Regarding the 49 indeterminate results, 43 (87.76%) were due to positive control failure, one (2.04%) due to negative control failure, and five (10.20%) due to both positive control and negative control failure. In the 30-day period prior to the QFT-Plus test, 46 (41.44%) of the study cohort were treated with immunotherapy (Table 1).

**Table 1:** Demographic and clinical characteristics

Variable	Median (IQR)
Age (year)	13 (11–14)
**Sex**	
Male Female	21 (18.92) 90 (81.08)
**Laboratory findings**	
White blood cells (10^9^/L)	6.14 (3.67–8.70)
Red blood cells (10^12^/L)	3.70 (3.13–4.17)
Hemoglobin (g/L)	103 (87–120)
Platelets (10^9^/L)	215 (153–263)
Neutrophils (10^9^/L)	4.42 (2.31–6.98)
Lymphocytes (10^9^/L)	1.13 (0.73–1.75)
Monocytes (10^9^/L)	0.37 (0.23–0.55)
**Blood biochemistry**	
Albumin (g/L)	30.70 (26-36.30)
A/G ratio	1.03 (0.79–1.40)
Urea (mmol/L)	8.42 (4.98–13.52)
Creatinine (µmol/L)	59.30 (43.20-90.83)
Total cholesterol (mmol/L)	4.48 (4.00-6.36)
Uric acid (µmol/L)	375.60 (271.20-503.25)
ALP (U/L)	85 (66–120)
AST/ALT	1.50 (1–2)
Total bilirubin (µmol/L)	7.56 (6.07–10.25)
Cholinesterase (U/L)	7576 (5228–10000)
LDH (U/L)	238 (186–315)
Triglycerides (mmol/L)	2.12 (1.69–3.32)
**Coagulation**	
Prothrombin time (second)	10.90 (10.20-11.59)
Fibrinogen (g/L)	2.94 (2.21–3.41)
Thrombin time (second)	17.30 (15.90–19.40)
APTT (second)	31.30 (27.10–35.60)
D-dimer (mg/L)	0.46 (0.19–0.82)
Antithrombin III activity (%)	112 (96–122)
**Complement system**	
Complement 3 (g/L)	0.38 (0.24–0.57)
Complement 4 (g/L)	0.06 (0.03–0.12)
**Early kidney injury biomarkers**	
Urinary IgG (mg/L)	106 (23.38-283.25)
Urinary microalbumin (mg/L)	804 (57.30–2360)
Urinary transferrin (mg/L)	88.90 (31.30–231)
Urinary α1-microglobulin (mg/L)	37.20 (18.55–94.65)
Urinary β2-microglobulin (µg/L)	510 (152.75-2666.25)
NAG (U/L)	21.45 (11.48–35.28)
**Erythrocyte sedimentation rate (mm/h)**	27 (12.50–61.50)
**Hospitalization frequency**	10 (3–15)
**SLEDAI-R**	13 (8–17)
	**N (%)**
**Co-morbidity**	
Respiratory disease	50 (45.05)
Cardiovascular disease	80 (72.07)
Hepatic disease	29 (26.13)
Nephropathy	30 (27.03)
Other autoimmune disorder	26 (23.42)
Multi-system disease	99 (89.19)
None	12 (10.81)
**Immunotherapy**	
Yes	46 (41.44)
No	65 (58.56)
**C-Reactive Protein**	
Abnormal	9 (8.11)
Normal	102 (91.89)
**QFT-Plus**	
Positive	2 (1.80)
Negative	60 (54.05)
Indeterminate	49 (44.14)

A/G: albumin/globulin ratio; ALP: alkaline phosphatase; AST/ALT: aspartate aminotransferase /alanine aminotransferase; LDH: lactate dehydrogenase; APTT: activated partial thromboplastin time; NAG: N-acetyl-β-D-glucosaminidase; SLEDAI-R: Systemic Lupus Erythematosus Disease Activity Index – Responder

### Univariate analysis of factors influencing indeterminate QFT-Plus results

Univariable logistic regression analysis indicated that the indeterminate group had significantly lower levels of hemoglobin, albumin, and ALP, fewer hospitalizations, and more frequent use of immunotherapy compared to the determinate group. In contrast, triglyceride and D-dimer levels were significantly elevated in the indeterminate group (Table 2).

**Table 2:** Univariate analyses of association with indeterminate QFT-Plus test

Variable	Determinate (*n* = 62)	Indeterminate (*n* = 49)	Odds Ratio (95% CI)	*P* value
	Median (IQR)	Median (IQR)		
Age	13 (11–14)	13 (11–14)	1.054 (0.907–1.226)	0.491
Male sex	12 (19.35)	9 (18.37)	0.938 (0.359–2.446)	0.895
**Laboratory findings**				
White blood cells (10^9^/L)	6.35 (4.65–8.17)	4.90 (3.27–9.55)	0.958 (0.859–1.069)	0.441
Hemoglobin (g/L)	115 (94.75–126)	95 (77-105.50)	0.968 (0.950–0.987)	0.001
Lymphocytes (10^9^/L)	1.34 (0.78–1.96)	1.07 (0.71–1.44)	0.705 (0.437–1.138)	0.152
**Blood biochemistry**				
Albumin (g/L)	34.40 (28.28–39.83)	29.10 (24.30-30.75)	0.866 (0.809–0.927)	<0.001
Creatinine (µmol/L)	50.10 (42.20–78)	69.90 (45.85-108.35)	1.001 (0.996–1.005)	0.784
ALP (U/L)	90.50 (70.75-145.24)	81 (60.50–111)	0.988 (0.98–0.997)	0.011
Triglycerides (mmol/L)	1.82 (1.30–2.38)	2.89 (2.11–3.95)	1.999 (1.382–2.891)	<0.001
**Coagulation**				
D-dimer (mg/L)	0.21 (0.10–0.49)	0.72 (0.46–1.10)	2.221 (1.214–4.064)	0.01
**Complement system**				
Complement 3 (g/L)	0.43 (0.31–0.71)	0.29 (0.22–0.42)	0.28 (0.07–1.122)	0.072
**Early kidney injury biomarkers**				
Urinary microalbumin (mg/L)	528 (32.95–1970)	1130 (156–2920)	1 (0.9999–1.0002)	0.714
**Erythrocyte sedimentation rate (mm/h)**	23 (12-48.25)	34 (20–69)	1.012 (0.999–1.026)	0.064
**Hospitalization frequency**	12 (6.75-17)	6 (2-12.50)	0.914 (0.861–0.971)	0.003
**SLEDAI-R**	12.5 (7–18)	14 (12–16)	1.052 (0.995–1.112)	0.077
	**N (%)**	**N (%)**		
**C-Reactive Protein**				
Normal	3 (4.84)	6 (12.24)	Reference	
Abnormal	59 (95.16)	43 (87.76)	2.744 (0.650-11.588)	0.170
**Immunotherapy**				
No	50 (80.65)	15 (30.61)	Reference	
Yes	12 (19.35)	34 (69.39)	9.444 (3.936–22.661)	<0.0001

ALP: alkaline phosphatase. SLEDAI-R: Systemic Lupus Erythematosus Disease Activity Index – Responder

### Multivariate analysis of factors influencing indeterminate QFT-Plus results

The final multivariate logistic regression analysis was shown in supplementary Tables 1, and the correlation matrix was performed to assure model integrity (supplementary Table 2). Moreover, multivariable logistic regression analysis revealed that triglycerides level (OR 1.847; 95% CI 1.195–2.856; *P* = 0.006), and immunotherapy use (OR 12.306; 95% CI 3.937–38.463; *P* < 0.0001) were independently associated with increased risk of indeterminate QFT-Plus results (Table 3).

**Table 3:** Multivariate analysis of predictors for an indeterminate QFT-Plus result

Variable	Odds Ratio	95% Confidence Interval	*P*-value
Erythrocyte sedimentation rate (mm/h)	1.007	0.986–1.029	0.501
Hemoglobin (g/L)	0.992	0.966–1.018	0.527
Albumin (g/L)	0.941	0.858–1.031	0.192
Triglycerides (mmol/L)	1.847	1.195–2.856	0.006
D-dimer (mg/L)	1.843	0.825–4.116	0.136
SLEDAI-R	0.970	0.892–1.055	0.473
Received immunotherapy	12.306	3.937–38.463	<0.0001
CRP abnormal	2.160	0.329–14.190	0.423

LN: lupus nephritis; CRP: C-reactive protein. SLEDAI-R: Systemic Lupus Erythematosus Disease Activity Index – Responder

### Cut-Off values for prediction of indeterminate QFT-Plus results

**Fig. 1:**
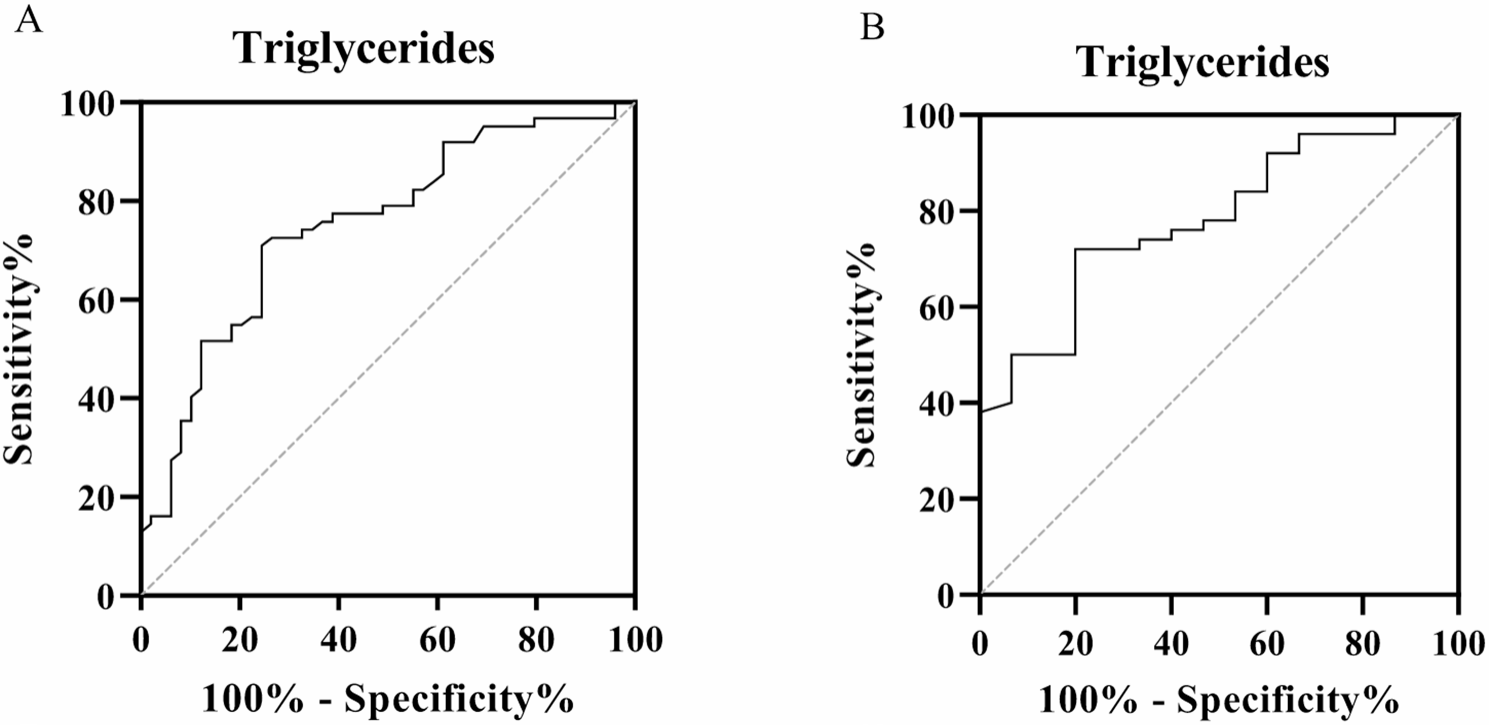
ROC curves illustrating the ability of triglyceride levels to discriminate indeterminate QFT-Plus results in all patients (**A**) and in patients who had not received immunotherapy (**B**)

ROC analysis was performed to evaluate the ability of triglyceride levels to predict indeterminate QFT-Plus results (Fig. 1; Table 4). At a cutoff value of 2.155 mmol/L, the area under the ROC curve was 0.751 (95% CI: 0.660–0.842; *P* < 0.0001), with a sensitivity of 70.97% and a specificity of 75.51% in all patients. Among patients who had not received immunotherapy, a cutoff of 2.210 mmol/L produced an AUC of 0.783 (95% CI: 0.663–0.904; *P* < 0.001), with corresponding sensitivity and specificity values of 72% and 73.33%, respectively.

**Table 4:** AUCs of triglycerides for discriminating indeterminate QFT-Plus results from determinate results

Variable	Population	AUC (95% CI)	*P* value	Std. Error	Youden’s index	Cut-off value	Sensitivity (%) (95% CI)	Specificity (%) (95% CI)
Triglycerides (mmol/L)	All patients	0.751 (0.660–0.842)	<0.0001	0.047	46.48	2.155	70.97 (58.71–80.78)	75.51 (61.91–85.40)
	Patients without immunotherapy	0.783 (0.663–0.904)	<0.001	0.061	45.33	2.210	72.00 (58.33–82.53)	73.33 (48.05–89.10)

### Patients with indeterminate QFT-Plus results with negative control failure

In total, six patients demonstrated indeterminate QFT-Plus results, including five cases with concurrent negative and positive control failures and one case with negative control failure. Notably, five of these patients (83.3%) were female. Laboratory investigations revealed consistent patterns of cytopenia and inflammatory activation: hemoglobin levels, serum albumin, and complement C3 levels were all subnormal, while acute-phase reactants including D-dimer and erythrocyte sedimentation rate were markedly elevated. Four patients with negative control failures were presented with concomittant hepatic dysfunction, and only one patient was naïve to immunotherapy (supplementary Table 3).

## Discussion

Indeterminate QFT-Plus results were associated with significant uncertainty when making a clinical decision on whether treatment for LTBI is required. Our study identified a remarkably high prevalence of indeterminate QFT-Plus results (44.14%) in pediatric LN, substantially exceeding the 3.0% rate previously reported in rheumatic disease populations [23]. Indeterminate results may occur due to two mechanisms: failure of the positive control tube with low IFN-γ production following phytohemagglutinin stimulation, or failure of the negative control tube with elevated IFN-γ levels in the unstimulated sample. Our analysis revealed that 87.76% indeterminate cases were due to positive control failure, reflecting LN-associated immune dysregulation. Excessive exposure of autoantigens in LN induces T-cell exhaustion, impairing Th1-mediated IFN-γ production and consequently compromising test sensitivity [24]. Two thirds of patients with negative control failures presented with concurrent hepatic dysfunction, which may stem from disease-associated systemic inflammation promoting nonspecific IFN-γ secretion - a phenomenon also documented in patients with malignancies, active TB, or metabolic disorders including hypertension and diabetes [25–27]. These findings suggest that the diverse underlying disease conditions may create an internal environment conducive to aberrant IFN-γ secretion. In addition to disease activity, numerous potential factors can also affect T-cell responses through multiple mechanisms, potentially compromising the reliability of IGRAs.

We revealed that immunotherapy agent usage, which may impair T cell activation, was an independent factor associated with the indeterminate QFT-Plus results in pediatric LN patients. However, the association between immunosuppressive agents and indeterminate IGRA results remains controversial across different clinical populations. While several studies involving immunocompromised or oncology patients have demonstrated this relationship with QFT-GIT results [28–31], a large-scale investigation of 408 rheumatic disease patients failed to establish this relationship [23]. These conflicting findings reflect several key variables, including fundamental differences in the underlying pathophysiology of various diseases, substantial heterogeneity in study population characteristics, and considerable variation in immunosuppressive regimens regarding drug class, dosage intensity, and treatment duration. Such methodological and clinical diversity across studies poses significant challenges in deriving consistent conclusions on attributable to the indeterminate IGRA result.

Additionally, elevated triglyceride levels were independently associated with indeterminate QFT-Plus results in our cohort. As a component of the triglyceride-glucose index, triglyceride may modulate immune cell function and polarization, potentially leading to the type of dysregulated immune responses that could contribute to an indeterminate assay outcome [32]. The role of triglyceride in TB immunopathology, however, is complex and seemingly paradoxical. For instance, while some studies associate higher baseline levels with TB treatment failure [33], others report lower triglyceride levels in active TB patients alongside more severe inflammation [34], highlighting a multifaceted relationship. Besides, triglycerides are linked to BMI, and underweight individuals with low levels are at higher TB risk [35]. Whereas our analysis revealed that triglycerides exhibited high predictive performance in patients not receiving immunotherapy, indicating their potential utility as immunodiagnostic biomarkers for predicting indeterminate QFT-Plus outcomes. These findings could help guide clinical decisions regarding the optimal timing of QFT-Plus testing in high-risk populations.

Our study has several limitations. First, due to the diversity of immunotherapy agents and limited sample size per agent, which could introduce selection bias and unreliable estimates if analyzed separately, we consolidated the original treatment categories into a binary variable. An expanded cohort will be enrolled to enable future stratified analyses of specific drug classes, doses, and treatment durations related to indeterminate QFT-Plus results. Second, the ROC analyses for triglycerides have not been subjected to internal and external validation. Finally, given the retrospective nature of this study and lack of systematic follow-up, we are unable to report the proportion of children with indeterminate QFT-Plus results who subsequently developed active TB. Future prospective studies incorporating larger sample sizes and systematic long-term follow-up will be conducted to address these issues.

## Conclusions

Hospitalized patients with LN had a high rate of an indeterminate QFT-Plus result, predominantly in positive control failure. Besides, our data showed that the use of immunotherapy agents and high triglycerides level represent factors associated with indeterminate QFT-Plus results in our cohort of pediatric lupus nephritis patients.

## Supplementary Information

Below is the link to the electronic supplementary material.

Supplementary Material 1

## Data Availability

The datasets used and/or analyzed during the current study available from the corresponding author on reasonable request.

## Abbreviations

TB: Tuberculosis
LTBI: Latent TB infection
MTB: Mycobacterium tuberculosis
SLE: Systemic lupus erythematosus
LN: Lupus nephritis
TST: Tuberculin skin test
IGRA: Interferon-gamma release assay
QFT-Plus: QuantiFERON-TB Gold Plus
ELISA: Enzyme-linked immunosorbent assay
nil: Negative-control
QFT-GIT: QuantiFERON-TB Gold In-Tube
WBC: Blood cells
RBC: Red blood cells
Hb: Hemoglobin
PLT: Platelets
A/G ratio: Albumin/globulin ratio
ALP: Alkaline phosphatase
AST/ALT: Aspartate aminotransferase/alanine aminotransferase
LDH: Lactate dehydrogenase
ESR: Erythrocyte sedimentation rate
PT: Prothrombin time
TT: Thrombin time
APTT: Activated partial thromboplastin time
AT-III: Antithrombin III activity
C3: Complement 3
C4: Complement 4
NAG: N-acetyl-β-D-glucosaminidase
IQR: Interquartile range
ORs: Odds ratios
CIs: Confidence intervals
