# Supplementary material for: Characteristics of indeterminate QuantiFERON-TB Gold PLUS results in pediatric lupus nephritis at Beijing Children’s Hospital, 2023–2024
Source: BMC Nephrol. 2025 Nov 25;27:9. doi: 10.1186/s12882-025-04652-9 (PMC12763920; doi:10.1186/s12882-025-04652-9)
Supplement: Supplementary file 1 — Supplementary Material 1 [file 12882_2025_4652_MOESM1_ESM.docx]

Supplementary table 1. Performance metrics of the multivariable logistic regression model

| Item | Elaboration |
| --- | --- |
| Working hypotheses | - The outcome variable is binary. - Observations are independent. - All included variables are independently associated with the primary outcome, and no substantial multicollinearity was detected. - A linear relationship exists between the independent variables and the logit of the outcome. |
| Handling of missing values | - Variables with a missing value rate of 5% or less were imputed using the group mean. - Variables exceeding a 5% missing rate were excluded from further analysis. |
| Variable selection | Multicollinearity was preliminarily assessed using correlation matrices and variance inflation factors. Variable selection incorporated univariate screening (*P* < 0.1), clinical relevance, and LASSO regression to prevent overfitting. |
| Selection of the model | The AIC was employed to facilitate model selection. |
| Discriminatory performance of the model | The discriminatory performance of the model was assessed using the C-statistic. |
| Overall evaluation of the final model | Akaike information criterion: 152.706 (intercept only) and 107.856 (intercept and covariates); likelihood ratio test: Chi-square= 60.8494, *P*< 0.0001; Wald test: Chi-square=30.8263, *P*= 0.0002 |
| Intercept | Estimate: -0.702; Standard error: 2.5357; Wald Chi-square: 0.0766; *P*=0.7819 |
| Validation of the predicted probabilities | Kendall’s Tau-α: 0.39; C-statistic: 0.893 |
| Method for calculating confidence intervals | Wald method |

Supplementary table 2. Spearman correlation matrix

| **Variable** | **WBC** | **Hemoglobin** | **Neutrophils** | **Lymphocytes** | **Monocytes** | **Albumin** | **Urea** | **Creatinine** | **ALP** | **Triglycerides** | **ESR** | **D_dimer** | **C3** | **C4** | **Uri_microalbumin** | **Urinary_2** |
| --- | --- | --- | --- | --- | --- | --- | --- | --- | --- | --- | --- | --- | --- | --- | --- | --- |
| **WBC** | 1 | 0.1268 | 0.94698 | 0.3797 | 0.65392 | -0.1272 | 0.06909 | -0.04131 | -0.02567 | 0.08377 | 0.03043 | -0.09687 | 0.2012 | 0.1491 | 0.13645 | -0.16171 |
| WBC |  | 0.1848 | <.0001 | <.0001 | <.0001 | 0.1834 | 0.4712 | 0.6668 | 0.7891 | 0.3821 | 0.7512 | 0.3141 | 0.0342 | 0.1235 | 0.1591 | 0.09 |
| **Hemoglobin** | 0.1268 | 1 | 0.05594 | 0.27934 | 0.21153 | 0.40749 | -0.44527 | -0.32912 | 0.33887 | -0.3568 | -0.24065 | -0.49012 | 0.4115 | 0.34282 | -0.12662 | -0.28165 |
| Hemoglobin | 0.1848 |  | 0.5598 | 0.003 | 0.0258 | <.0001 | <.0001 | 0.0004 | 0.0003 | 0.0001 | 0.011 | <.0001 | <.0001 | 0.0003 | 0.1916 | 0.0027 |
| **Neutrophils** | **0.94698** | 0.05594 | 1 | 0.15964 | 0.57045 | -0.15144 | 0.12534 | 0.02774 | -0.14477 | 0.10311 | 0.04867 | -0.03274 | 0.20098 | 0.11175 | 0.17989 | -0.10832 |
| Neutrophils | <.0001 | 0.5598 |  | 0.0942 | <.0001 | 0.1126 | 0.1899 | 0.7726 | 0.1295 | 0.2815 | 0.612 | 0.7342 | 0.0344 | 0.2495 | 0.0625 | 0.2578 |
| **Lymphocytes** | 0.3797 | 0.27934 | 0.15964 | 1 | 0.40356 | -0.02128 | -0.15156 | -0.21772 | 0.28477 | -0.05361 | 0.10819 | -0.12289 | 0.04438 | 0.06421 | 0.01563 | -0.19774 |
| Lymphocytes | <.0001 | 0.003 | 0.0942 |  | <.0001 | 0.8246 | 0.1123 | 0.0217 | 0.0025 | 0.5763 | 0.2583 | 0.2009 | 0.6437 | 0.5092 | 0.8724 | 0.0375 |
| **Monocytes** | **0.65392** | 0.21153 | **0.57045** | 0.40356 | 1 | 0.0042 | -0.09138 | -0.064 | 0.03019 | 0.08758 | 0.02479 | -0.12009 | 0.28868 | 0.13817 | 0.01605 | -0.06977 |
| Monocytes | <.0001 | 0.0258 | <.0001 | <.0001 |  | 0.9651 | 0.3402 | 0.5045 | 0.7531 | 0.3607 | 0.7962 | 0.2114 | 0.0021 | 0.1539 | 0.8691 | 0.4668 |
| **Albumin** | -0.1272 | 0.40749 | -0.15144 | -0.02128 | 0.0042 | 1 | -0.43002 | -0.31305 | 0.15345 | -0.4697 | -0.39415 | -0.59468 | 0.45934 | 0.36788 | -0.40164 | 0.02967 |
| Albumin | 0.1834 | <.0001 | 0.1126 | 0.8246 | 0.9651 |  | <.0001 | 0.0008 | 0.1078 | <.0001 | <.0001 | <.0001 | <.0001 | <.0001 | <.0001 | 0.7572 |
| **Urea** | 0.06909 | -0.44527 | 0.12534 | -0.15156 | -0.09138 | -0.43002 | 1 | 0.75406 | -0.18767 | 0.52744 | 0.19061 | 0.37134 | -0.31277 | -0.27476 | 0.35433 | -0.16441 |
| Urea | 0.4712 | <.0001 | 0.1899 | 0.1123 | 0.3402 | <.0001 |  | <.0001 | 0.0486 | <.0001 | 0.0451 | <.0001 | 0.0008 | 0.004 | 0.0002 | 0.0847 |
| **Creatinine** | -0.04131 | -0.32912 | 0.02774 | -0.21772 | -0.064 | -0.31305 | **0.75406** | 1 | -0.13258 | 0.26777 | 0.1577 | 0.3084 | -0.15997 | -0.17834 | 0.36422 | -0.14535 |
| Creatinine | 0.6668 | 0.0004 | 0.7726 | 0.0217 | 0.5045 | 0.0008 | <.0001 |  | 0.1654 | 0.0045 | 0.0983 | 0.001 | 0.0935 | 0.0648 | 0.0001 | 0.128 |
| **ALP** | -0.02567 | 0.33887 | -0.14477 | 0.28477 | 0.03019 | 0.15345 | -0.18767 | -0.13258 | 1 | -0.12205 | -0.00879 | -0.18264 | 0.07548 | 0.08825 | -0.208 | -0.14978 |
| ALP | 0.7891 | 0.0003 | 0.1295 | 0.0025 | 0.7531 | 0.1078 | 0.0486 | 0.1654 |  | 0.2019 | 0.9271 | 0.0562 | 0.4311 | 0.3637 | 0.0308 | 0.1166 |
| **Triglycerides** | 0.08377 | -0.3568 | 0.10311 | -0.05361 | 0.08758 | -0.4697 | **0.52744** | 0.26777 | -0.12205 | 1 | 0.16401 | 0.27674 | -0.2662 | -0.12828 | 0.23545 | 0.03289 |
| Triglycerides | 0.3821 | 0.0001 | 0.2815 | 0.5763 | 0.3607 | <.0001 | <.0001 | 0.0045 | 0.2019 |  | 0.0854 | 0.0034 | 0.0047 | 0.1858 | 0.0142 | 0.7318 |
| **ESR** | 0.03043 | -0.24065 | 0.04867 | 0.10819 | 0.02479 | -0.39415 | 0.19061 | 0.1577 | -0.00879 | 0.16401 | 1 | 0.39948 | -0.19271 | -0.14113 | 0.09315 | 0.12405 |
| ESR | 0.7512 | 0.011 | 0.612 | 0.2583 | 0.7962 | <.0001 | 0.0451 | 0.0983 | 0.9271 | 0.0854 |  | <.0001 | 0.0427 | 0.1452 | 0.3376 | 0.1946 |
| **D_dimer** | -0.09687 | -0.49012 | -0.03274 | -0.12289 | -0.12009 | -0.59468 | 0.37134 | 0.3084 | -0.18264 | 0.27674 | 0.39948 | 1 | -0.332 | -0.29143 | 0.26909 | 0.15091 |
| D_dimer | 0.3141 | <.0001 | 0.7342 | 0.2009 | 0.2114 | <.0001 | <.0001 | 0.001 | 0.0562 | 0.0034 | <.0001 |  | 0.0004 | 0.0023 | 0.0051 | 0.1156 |
| **C3** | 0.2012 | 0.4115 | 0.20098 | 0.04438 | 0.28868 | 0.45934 | -0.31277 | -0.15997 | 0.07548 | -0.2662 | -0.19271 | -0.332 | 1 | 0.76017 | -0.08007 | 0.07043 |
| C3 | 0.0342 | <.0001 | 0.0344 | 0.6437 | 0.0021 | <.0001 | 0.0008 | 0.0935 | 0.4311 | 0.0047 | 0.0427 | 0.0004 |  | <.0001 | 0.4101 | 0.4626 |
| **C4** | 0.1491 | 0.34282 | 0.11175 | 0.06421 | 0.13817 | 0.36788 | -0.27476 | -0.17834 | 0.08825 | -0.12828 | -0.14113 | -0.29143 | **0.76017** | 1 | -0.2195 | -0.00964 |
| C4 | 0.1235 | 0.0003 | 0.2495 | 0.5092 | 0.1539 | <.0001 | 0.004 | 0.0648 | 0.3637 | 0.1858 | 0.1452 | 0.0023 | <.0001 |  | 0.0245 | 0.9211 |
| **Uri_microalbumin** | 0.13645 | -0.12662 | 0.17989 | 0.01563 | 0.01605 | -0.40164 | 0.35433 | 0.36422 | -0.208 | 0.23545 | 0.09315 | 0.26909 | -0.08007 | -0.2195 | 1 | 0.10437 |
| Uri_microalbumin | 0.1591 | 0.1916 | 0.0625 | 0.8724 | 0.8691 | <.0001 | 0.0002 | 0.0001 | 0.0308 | 0.0142 | 0.3376 | 0.0051 | 0.4101 | 0.0245 |  | 0.2824 |
| **Urinary_2** | -0.16171 | -0.28165 | -0.10832 | -0.19774 | -0.06977 | 0.02967 | -0.16441 | -0.14535 | -0.14978 | 0.03289 | 0.12405 | 0.15091 | 0.07043 | -0.00964 | 0.10437 | 1 |
| Urinary_2 | 0.09 | 0.0027 | 0.2578 | 0.0375 | 0.4668 | 0.7572 | 0.0847 | 0.128 | 0.1166 | 0.7318 | 0.1946 | 0.1156 | 0.4626 | 0.9211 | 0.2824 |  |

The numbers in the first and second lines for each parameter were spearman correlation coefficients and *P*-value, respectively.

Supplementary table 3. Description of patient characteristics with indeterminate QFT-Plus results with negative control failure

| Variable | Patient | | | | | |
| --- | --- | --- | --- | --- | --- | --- |
|  | #1 | #2 | #3 | #4 | #5 | #6 |
| Age | 12 | 13 | 10 | 12 | 13 | 13 |
| Sex | F | F | M | F | F | F |
| IFN-γ level in Nil tube | 48.79 | 9.81 | 29.15 | 8.34 | 10.27 | 50.98 |
| IFN-γ level in Mitogen tube | 0 | 0 | 0 | 1.41 | 0 | 0 |
| **Laboratory findings** |  |  |  |  |  |  |
| White blood cells (10^9^/L) | 1.98^a^ | 7.29 | 2.93^a^ | 9.7 | 3.66^a^ | 2.53^a^ |
| Hemoglobin (g/L) | 73^a^ | 62^a^ | 104^a^ | 103^a^ | 106^a^ | 104^a^ |
| Neutrophils (10^9^/L) | 0.9^a^ | 5.54 | 2.38 | 7 | 2.08 | 1.14^a^ |
| Lymphocytes (10^9^/L) | 0.84^a^ | 1.39^a^ | 0.37^a^ | 1.96 | 1.29^a^ | 1.1^a^ |
| Monocytes (10^9^/L) | 0.23 | 0.34 | 0.18 | 0.74 | 0.28 | 0.23 |
| **Blood biochemistry** |  |  |  |  |  |  |
| Albumin (g/L) | 21.8^a^ | 27^a^ | 26.5^a^ | 23.3^a^ | 24.3^a^ | 37.6^a^ |
| Urea (mmol/L) | 11.67^b^ | 13.52^b^ | 4.16 | 10.92^b^ | 13.93^b^ | 2.42^a^ |
| Creatinine (µmol/L) | 108.1^b^ | 96.4^b^ | 34.6 | 67.8^b^ | 97.8^b^ | 35.3 |
| ALP (U/L) | 120 | 25 ^a^ | 66 ^a^ | 90 | 70 ^a^ | 116 |
| Triglycerides (mmol/L) | 2.37^b^ | 2.89^b^ | 1.68 | 3.61^b^ | 2.2^b^ | 1.41 |
| **Coagulation** |  |  |  |  |  |  |
| D-dimer (mg/L) | 2.481^b^ | 0.859^b^ | 0.787^b^ | 1.043^b^ | 0.705^b^ | 0.412^b^ |
| **Complement system** |  |  |  |  |  |  |
| Complement 3 (g/L) | 0.13^a^ | 0.26^a^ | 0.54^a^ | 0.82^a^ | 0.29^a^ | 0.71^a^ |
| Complement 4 (g/L) | 0.022^a^ | 0.03^a^ | 0.18 | 0.1^a^ | 0.07^a^ | 0.104^a^ |
| **Early kidney injury biomarkers** |  |  |  |  |  |  |
| Urinary microalbumin (mg/L) | 804^b^ | 24.7^b^ | 156^b^ | 1370^b^ | 683^b^ | 2.01 |
| Urinary β2-microglobulin (µg/L) | 532^b^ | 13499^b^ | 924^b^ | 691^b^ | 40 | 176^b^ |
| Erythrocyte sedimentation rate (mm/h) | 34^b^ | 140^b^ | 38.76^b^ | 38.76^b^ | 59^b^ | 63^b^ |
| Length of stay | 19 | 1 | 1 | 5 | 4 | 1 |
| Hospitalization frequency | 2 | 12 | 5 | 3 | 2 | 13 |
| Co-morbidity | Lupus encephalopathy,  Hyperkalemia, Hypertension Myocardial injury,  Hepatic dysfunctio,  Pleural effusion,  Ascites,  Moderate anemia,  Respiratory tract infection | Autoimmune Hemolytic Anemia, Respiratory Tract Infection | Leukopenia, Mild Anemia, Hepatic Dysfunction,  Hepatic Steatosis | Acute Kidney Injury, Thrombotic Microangiopathy, Pulmonary Involvement, Pulmonary Edema, Pericardial Effusion, Nutcracker Phenomenon | Hepatic Dysfunction | Antiphospholipid Syndrome, Hepatic Dysfunction, Respiratory Tract Infection, Coagulation Disorder |
| Therapy | γ-globulin | Methylprednisolone, Rituximab | γ-globulin | None | γ-globulin | γ-globulin |

a: below the lower limit of normal; b: above the upper limit of normal
